# Effects of the Probiotics *Lactobacillus rhamnosus* DM163 and *Lactobacillus delbrueckii* DPUL‐F36 on Anxiety‐Related and Emotional Behaviors in Male Wistar Rats Under an Altered Light–Dark Cycle

**DOI:** 10.1002/brb3.70814

**Published:** 2025-09-11

**Authors:** Farnaz Ghayour Babaei, Ali Moghimi, Ehsan Saburi, Ali Makhdoumi, Morteza Behnam Rasouli

**Affiliations:** ^1^ Department of Biology, Faculty of Science Ferdowsi University of Mashhad Mashhad Iran; ^2^ Rayan Research Center For Neuroscience and Behavior, Department of Biology, Faculty of Science Ferdowsi University of Mashhad Mashhad Iran; ^3^ Medical Genetics Research Center Mashhad University of Medical Sciences Mashhad Iran

**Keywords:** Anxiety, light–dark cycle, microbiota, probiotics

## Abstract

**Introduction:**

Anxiety and stress are prevalent mental health issues. Traditional drug treatments often come with unwanted side effects and may not produce the desired results. As an alternative, probiotics are being used as a treatment option due to their lack of specific side effects. Probiotics are live microorganisms that, in sufficient amounts, benefit the host's health. The human digestive tract naturally contains hundreds of different types of bacteria; among these bacteria, probiotic strains of *Lactobacillus rhamnosus* DM163 and *Lactobacillus delbrueckii* DPUL‐F36 are among the species that may improve behavioral disorders such as anxiety and stress.

**Methods:**

In this study, 42 adult male Wistar rats (190–220 gr/BW), categorized into six groups, were used. Except for the control group, the rest were subjected to 30 days of light–dark cycle alteration (4 h dark, 20 h light). Simultaneously with the light/dark disruption, these groups were gavaged daily doses of 1 × 10^9^ colony‐forming units (CFU) of the target probiotics. Stool samples were collected to confirm changes in the gut microbiota before and after administration of probiotics. Following DNA extraction, PCR was performed using specific primers. The open‐field test and the elevated plus maze test were used to check the anxiety, stress, and exploratory behaviors.

**Results:**

The comparison of behavioral tests showed that the change in the light–dark cycle caused negative behavioral changes, and the administration of probiotics, particularly *L. rhamnosus*, was found to be more effective in reducing anxiety and stress levels and improving exploratory behavior compared to *L. delbrueckii*. The qualitative PCR test also determined that during the 30‐day intervention period, *L. rhamnosus* and *L. delbrueckii* bacteria were present in the intestinal bacterial flora of rats.

**Discussion:**

Changes in the light–dark cycle cause significant disturbances in normal physiology. These alterations are especially evident in the functions of the central nervous system and various behaviors. So, in the long term, it can seriously lead to destructive neurodegenerative alterations of the nervous system. Probably, the increase and predominance of the population of probiotics and the effects of their metabolites using the gut–brain axis will lead to beneficial and even preventive effects.

## Graphical abstract

The systematic experimental protocol detailed in this graphical abstract demonstrates the methodology used to assess the impact of probiotics on rat behavior and gut microbiota, integrating behavioral tests with molecular analysis.

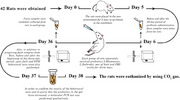

## 1 Introduction

Anxiety is a chronic and intense experience of fear regarding an imminent event and affects approximately 29% of humans during their lifetime (Halgin and Whitbourne 2003). Among the methods for managing anxiety is the use of drugs; however, these drugs are ineffective in almost one‐third of people suffering from anxiety and stress. Moreover, most of these drugs have side effects of nausea, vomiting, headache, sexual dysfunction, and weight gain (Ionescu and Papakostas 2017).

Today, the digestive system has been proposed as a target for new therapeutic interventions to improve various behavioral disorders, including anxiety, stress, and depression, as well as many neurodegenerative diseases such as Alzheimer's and Parkinson's. This is due to the complex communication network between these conditions and the gut microbiota. In the large intestine, a population of beneficial bacteria, known as probiotics, can exert beneficial effects when ingested via oral administration (Mokhtari et al. 2017). Several studies have explored the positive effects of probiotics on human and rat health, including reducing anxiety symptoms and improving exploratory behaviors (Rahman et al. 2017). Probiotics are important for enhancing neurological health due to their anti‐inflammatory properties. Overall, findings indicate the positive effect of probiotics in improving these behaviors without serious side effects (Park et al. 2018).

The potential mechanisms by which probiotics influence emotional behaviors are multifaceted and involve complex interactions between the gut microbiome, the immune system, and the gut–brain axis. The gut–brain axis, a bidirectional communication network between the gastrointestinal tract and the central nervous system, plays a key role through the vagus nerve, immune pathways, and microbial interactions that can influence neurotransmitters and hormones related to anxiety and stress (Cryan and Dinan 2012). Additionally, some probiotics can produce bioactive metabolites, such as short‐chain fatty acids (SCFAs), which possess neuroprotective properties and may mitigate oxidative processes exacerbated by chronic light–dark stress. Prior studies have indicated that these metabolites might exhibit significant antioxidative effects (Dalile et al. 2019). Moreover, probiotics can affect the production and metabolism of neurotransmitters like serotonin, dopamine, and gamma‐aminobutyric acid (GABA), which are crucial in mood regulation and anxiety (Strandwitz 2018).

The central nervous system structures, such as the hypothalamus and pituitary–adrenal axis, along with the vagus nerve (which transmits information between the intestine and brain) and the GABAergic system (the most important inhibitory neurotransmitter of the CNS), are the key pathways that play a role in the physiological processes of anxiety (Kane and Kinzel 2018; Slykerman et al. 2017).

Probiotics present in the gut microbiome play a role in the production of tryptophan (a serotonin precursor), thereby enhancing serotonin signaling and improving the symptoms of stress, anxiety, and depression (Kennedy et al. 2017).

One of the underlying factors contributing to anxiety and stress is disruption in the sleep cycle, which can occur with alterations of the light–dark cycle. Nowadays, humans are frequently exposed to unnaturally bright light at night, causing serious disruptions to biological systems. This disruption can lead to metabolic and biochemical changes in human physiological systems. The problem of light at night has been exacerbated by urban development. This “light pollution” currently affects 99% of the population in the United States and Europe. Electric lights have created light pollution and disturbed human sleep and wakefulness patterns (Navara and Nelson 2007).

In general, people who are exposed to light at night face an increased risk of various health issues, including heart complications (Ha and Park 2005), cancer (Davis and Mirick 2006), sleep alterations (Kohyama 2009), circadian cycle alterations (Borugian et al. 2005), mood alterations (Dumont and Beaulieu 2007), and reproductive dysfunctions (Thomas et al. 2001).

Exposure to light increases the concentration of corticosterone. Nocturnal light exposure may deleteriously affect animals directly by disrupting their biological clock (Ohta et al. 2005). Another possibility is that exposure to light at night represents a chronic stressor that can indirectly affect physiological and behavioral processes (Ma et al. 2007).

As mentioned above, today's altered human lifestyle, especially the use of high‐density and long‐term exposure to light during the dark cycle of the circadian rhythm, and the impact of the digestive system, which continuously sends biochemical signals to the brain via the gut–brain axis, may provide new insights into the physiological effects of diets on behaviors. Another important aspect of this study is the idea of the effect of lifestyle (light exposure) on neurodegenerative diseases such as Alzheimer's, which will enhance our understanding of the pathophysiology of such disorders.

In this study, we tried to show that adding probiotics to the diet of rats can improve emotional behaviors such as anxiety, stress, and exploratory behaviors.

## 2 Materials and Methods

### 2.1 Animals

#### 2.1.1 Ethics Statement

The animal experimental protocol was conducted with the approval of the ethics committee of the Ferdowsi University of Mashhad (code: IR.UM.REC.1401.080). All procedures in the animal experiments were strictly followed by the guidelines for the care and use of laboratory animals issued by the National Institutes of Health.

#### 2.1.2 Model Establishment

A total of 42 healthy adult male Wistar rats (with an average weight of 210 ± 10 gr) were purchased from the Laboratory Animal Care Center of Mashhad University of Medical Sciences (Mashhad, Iran). Male rats were selected for this study based on the findings of Scholl et al. (2019) and Domonkos et al. (2017), which indicate that stress impacts female rats less significantly than males. Moreover, hormonal fluctuations associated with the estrous cycle in female rats can influence anxiety and emotional behaviors, potentially introducing variability in results (Singh et al. 2023). Using male rats helps avoid these hormonal effects and ensures greater stability in experimental conditions. Male Wistar rats are also more frequently used in similar studies, providing better comparability with previous research.

Rats were placed in clean rectangular polypropylene cages. Water and food for laboratory rodents were available ad libitum until the end of the experiment. The cages were housed in a room under controlled conditions at 22 ± 1°C with a 12 h:12 h light/dark cycle.

**FIGURE 1:**
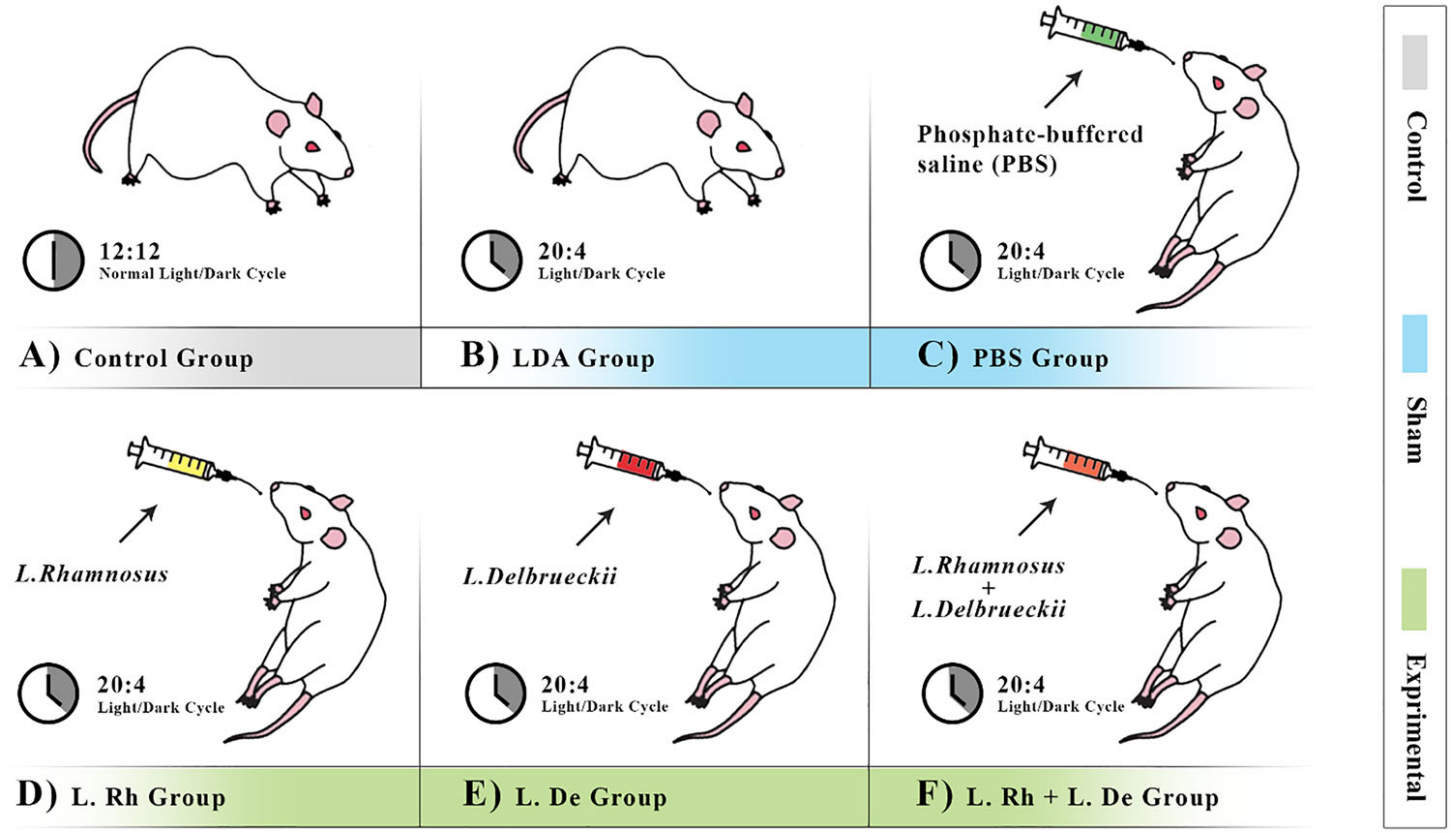
Grouping of rats based on light–dark cycle alterations (LDA) and probiotics administration. (A) Control: without administration of any substance and LDA. (B) Sham‐1: without administration of any substance and undergoing LDA. (C) Sham‐2: administration of PBS solution and undergoing LDA. (D) Exprimental‐1: administration of *L. rhamnosus* probiotic (L. Rh) and undergoing LDA. (E) Exprimental‐2: administration of *L. delbrueckii* Probiotic (L. De) and undergoing LDA. (F) Exprimental‐3: administration of combined two *L. rhamnosus* and *L. delbrueckii* probiotics (L. Rh + L. De) and undergoing LDA.

After 1 week of adaptation, the rats, based on alterations in the light–dark cycle (LDA) and administration of probiotics, were randomly arranged into six groups (*n* = 7 rats/group): one group as Control, two groups as Sham, and three groups as Experimental (Figure 1).

Ultimately, the strategic inclusion of these sham groups is essential for disentangling the contributions of each independent variable to the observed outcomes in our study, providing valuable insights into the effects of probiotics on anxiety and emotional behaviors in male Wistar rats under altered light–dark conditions.

Sham and experimental groups’ cages (seven rats were housed per cage) were placed in a separate room for 30 days. This room was under conditions at 22 ± 1°C with a 4 h:20 h light/dark cycle (4 h of light and 20 h of dark). We have some other yet unpublished studies (dissertations) using many aspects of this light–dark alteration method that illustrated the impact of such a cycle on different behaviors. The lighting of this room was provided by a lamp (150–200 lux), which automatically controlled the on and off and lighting hours using a digital timer. This light intensity range of 150–200 lux is consistent with previous studies that have investigated the effects of altered light–dark cycles in rodents, including Wistar rats (Stenvers et al. 2016). The timer was set to keep the lamp off from 3:00 a.m. to 7:00 a.m. (4 h) and on for the remaining 20 h of the day and night.

The control group's room condition was the same but with a normal light/dark cycle (12 h:12 h).

### 2.2 Probiotics

Based on the studies of Olorocisimo et al. (2023); Lin et al. (2022), and Schmidt et al. (2024), which showed positive effects on improving inflammation, anxiety, and stress, two strains of bacteria, “*Lactobacillus delbrueckii* DPUL‐F36” and “*Lactobacillus rhamnosus* DM163,” purchased from the Iranian Biological Resource Center (IBRC, Tehran, Iran), were used in this research.

For each round of gavage, bacterial cells were inoculated into MRS broth medium in a 15 mL Falcon tube and put in a candle jar (anaerobic environment). Then the candlestick was placed in the incubator at 37°C. After 12 h, the bacterial cells were harvested following centrifugation at 5000 rpm for 15 min. The pellets were resuspended in sterile PBS buffer and counted using a spectrophotometer (OD_620 nm_).

A total of 10^9^ CFU were used by gavage for daily consumption. This dosage selection is supported by studies such as Latif et al. (2023), which highlight the effectiveness of high CFU counts in achieving significant biological impacts.

A small‐sized gavage needle was used, and the solution after each preparation was directly introduced into the rat's stomach. During gavage, gloves were changed, and to prevent contamination, the environment was cleaned regularly. Also, the needles were completely sterilized after each use. Gavage was carried out according to the ethical guidelines for working with laboratory animals, and we tried to put the rats under the least amount of stress.

### 2.3 Molecular Confirmation of Bacterial Settlement

#### 2.3.1 DNA Extraction

The DNA needed to perform the molecular test was extracted from the feces of the rats of the groups listed in Table 1. For this purpose, the DNA was extracted using a commercial tissue genomic DNA extraction kit (Pars‐Tous Co. IRAN) according to the protocol. Also, to improve the quality of extracted DNA, a heating method (incubation at 95°C for 15 min) was used to remove confounding factors before extraction. The DNA quantity and quality (A260/A280 and A260/A230) were measured using a spectrophotometer (NanoDrop One, Thermo Scientific), and the DNA was stored at −20°C until use.

**TABLE 1:** Quantitative content of administration substances and alterations made in the light/dark cycle.

Period	Groups	Probiotics (CFU)		PBS (cc)	Light–dark alterations (LDA)
		*L. delbrueckii*	*L. rhamnosus*		
30 Days−	Control	−	−	−	−
+	LDA	−	−	−	+
+	PBS	−	−	+	+
+	L. Rh	+	−	−	+
+	L. De	−	+	−	+
+	L. Rh + L. De	+	+	−	+

#### 2.3.2 Quantitative Examination of Extracted DNA Using a Nanodrop Device

The amount of extracted DNA was measured by the Nanodrop device. This device determined the concentration and quality of DNA based on the wavelength passing through the target sample. In this method, the qualitative characteristics of DNA were measured by reading the OD_260_/OD_280_ ratio, in which the amount of light absorption for DNA was measured at a wavelength of 260 nm, and the amount of DNA was shown. The samples in this research included the control group and the two experimental groups, L. Rh and L. De, and after examination, their OD_260_/OD_280_ was between 1.84 and 2.07.

#### 2.3.3 Determining and Checking Primer Sequences

**FIGURE 2:**
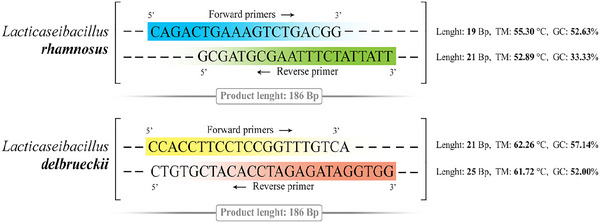
Sequence of primers used in this research.

The desired primer sequences were selected after reading various articles and then searched using the NCBI website (https://www.ncbi.nlm.nih.gov/) and checked for cross‐homology with repetitive sequences or other locations in the genome because such homology can lead to false primes and the production of nonspecific amplicons. The primer sequences that were synthesized by Pishgaman Biotechnology Co. (Tehran, Iran) are depicted in Figure 2.

#### 2.3.4 PCR

After sterilizing the equipment in the autoclave, the required materials and equipment were placed into the PCR workstation for 10–15 min to be disinfected. Then, as per the tables below, the materials were combined under the hood and then placed inside the PCR machine (Tables 2 and 3). Before putting the solution inside the PCR machine, the microtubes were spun so that the solution particles did not stick to the wall and head of the microtube.

**TABLE 2:** Necessary materials to perform qualitative PCR.

Materials	Volume (μ L)
Deionized water	4.5
DNA	2
Taq master mix	7.5
Specific Primer (Forward)	0.5
Specific Primer (Reverse)	0.5
Total	15

**TABLE 3:** Thermocycler PCR protocol for Phusion HF polymerase.

Steps	Temperature	Time (sec)	Cycles
Initial denaturation	94°C	300	1
Denaturation	94°C	30	35
Annealing	58°C	30	
Extension	72°C	30	
Final extension	72°C	300	1

### 2.4 Behavioral Tests

Rats were moved to the testing room at least 30 min before all behavioral experiments. During the interval between events, the test equipment was regularly cleaned with 70% ethyl alcohol to avoid olfactory cues. Behavioral tests included the Open‐Field (OF) and the Elevated Plus Maze (EPM). The OF is a widely used and validated tool for evaluating locomotor activity and anxiety‐related behaviors in experimental studies, and the EPM is a well‐established behavioral test widely used for evaluating anxiety in rodents.

#### 2.4.1 OF Test

The behaviors of rats were recorded by a camera while freely moving in a 100 × 100 cm^2^ black square flat arena for 5 min; the path followed by the animals was tracked and analyzed by using the Any‐maze software version 7.2 (Stoelting, Wood Dale, IL, USA).

The measured behavioral parameters included total distance moved (traveled) in the central (inner) arena, time spent in the inner zone, and total mobile time in the inner zone. The amount of time spent in the center of the OF was considered an indication of reduced stress in the rat, while time spent in the perimeter suggested higher stress levels. Furthermore, a greater distance traveled inside the box, particularly in the center, was indicative of higher exploratory activities in the rat (Prut and Belzung 2003; Walsh and Cummins 1976).

#### 2.4.2 Elevated Plus Maze Test

The apparatus was 50 cm above the floor and had a center zone (10 cm°×°10 cm), two closed arms (CA) with walls (100 cm × 10 cm ×30 cm), and two open arms (OA) (100 cm×10 cm). Rats were placed at the center facing the CA and freely explored for 5 min. The percent of time spent, the percent of entries, the total traveled distance, mobility time, average speed, mean visit, and total time freezing in the open and enclosed arms are recorded and analyzed by Any‐maze software version 7.2 (Stoelting, Wood Dale, IL, USA).

In our study, the OF was exclusively used to assess exploratory behaviors, while both the OF and the EPM were employed for a comprehensive evaluation of anxiety and stress. This dual approach allowed us to differentiate between exploratory behaviors and anxiety‐related responses effectively. (Walf and Frye 2007; Pellow et al. 1985).

### 2.5 Statistical Analyses

Data were analyzed using a one‐way analysis of variance (ANOVA) followed by a post hoc Tukey test to determine the statistical significance between groups. All data are presented as the mean ± SEM, and values of *p* < 0.05 indicate statistical significance.

**FIGURE 3:**
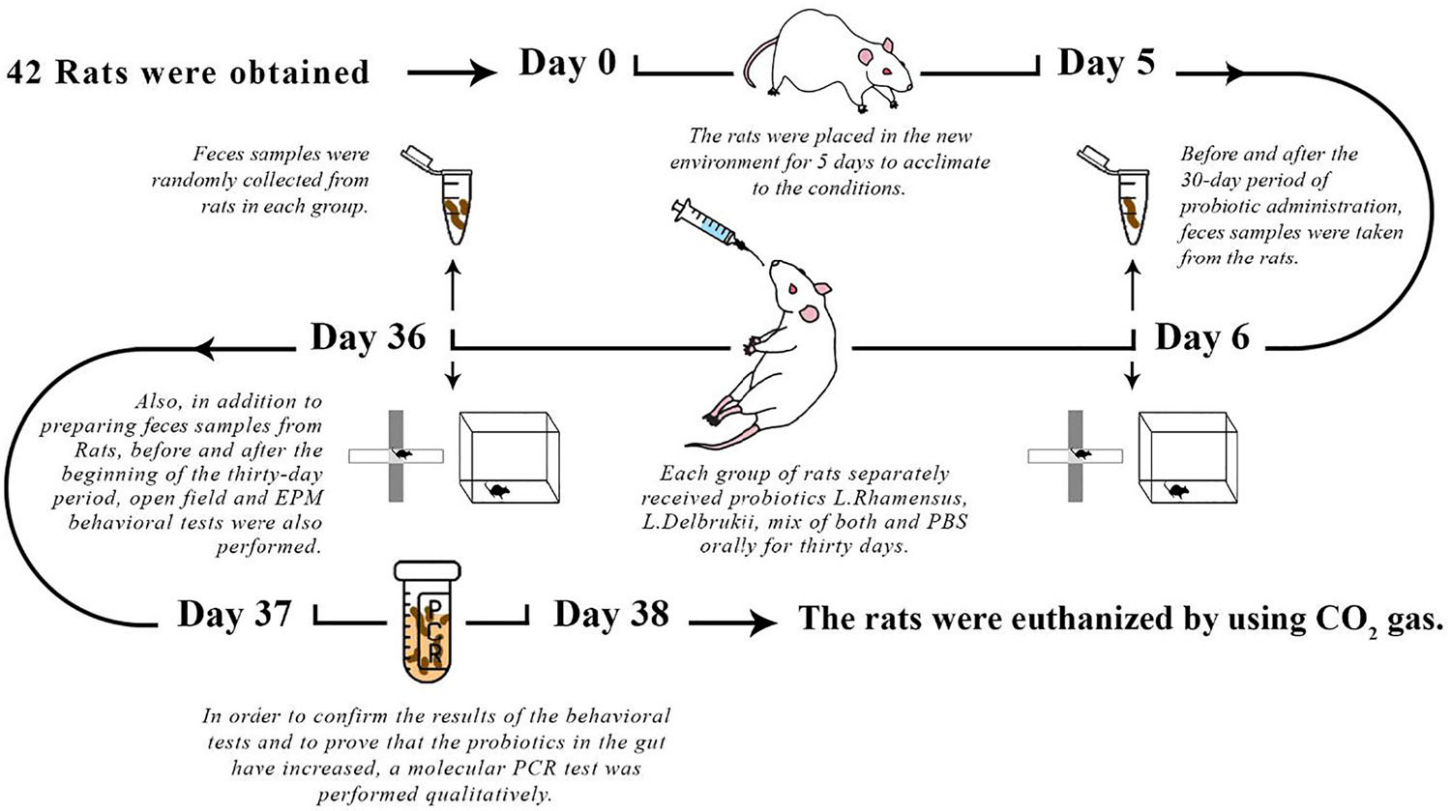
A schematic representation of the administration and test steps.

The data were organized and analyzed with the Prism 9.0.0 software (GraphPad, Boston, MA, USA). The following figure is schematic representaion of all methological plan and stages used in this study (Figure 3).

## 3 Results

### 3.1 Impact of Altering the Light–Dark Cycle on Emotional and Exploratory Behaviors

**FIGURE 4:**
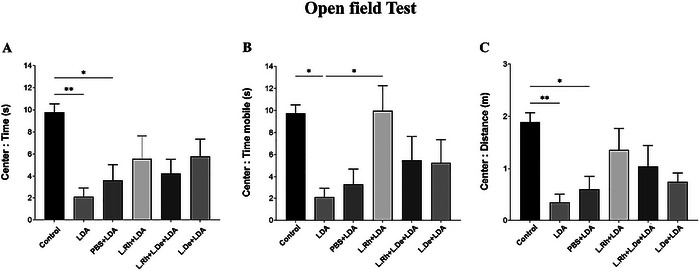
Behavioral response in an OF test. (A) Time spent in the central zone. (B) The total mobility time in the central areas. (C) The total distance (traveled) in the central arena. (Histogram bars indicate the mean ± SEM. A *p* < 0.05 was considered significant. *p < 0.05, **p < 0.01, ***p < 0.001).

During the OF test, rats in the LDA group spent significantly less time and covered less distance in the central area compared to the control group (*p* < 0.01) (Figure 4A,C). Also, the LDA group was mobile for a shorter duration compared to the L. Rh + LDA and LDA groups in the central area, and this difference between them was significant (*p* < 0.05) (Figure 4B).

**FIGURE 5:**
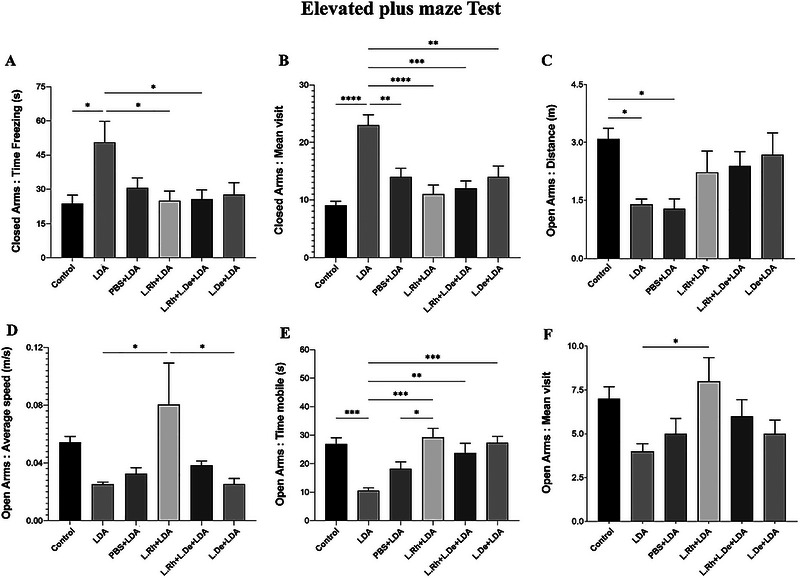
Behavioral response in an EPM test. (A) Total inactive time (freezing) in CA. (B) The mean visiting number of the CA. (C) The total travel distance in OA. (D) The rat's average speed in OA. (E) The total moving time (mobility) in OA. (F) The mean number of OA visits. (The results for the data were expressed as the mean ± SEM. A *p* < 0.05 was considered significant. *p < 0.05, **p < 0.01, ***p < 0.001).

In the EPM test, rats in the LDA group remained frozen longer in the CA than those in the control group, as well as those in the L. Rh + LDA and L. Rh + L. De + LDA groups (*p* < 0.05) (Figure 5A). Furthermore, results from the EPM test indicated that the LDA group had significantly more visits to the CA compared to the other groups (Figure 5B).

This is also evident in the parameters of distance traveled, average speed, time mobile, and mean visit to OA (Figure 5C–F). These findings suggest that alterations in the light–dark cycle in rats are associated with increased anxiety levels and diminished exploratory behaviors.

### 3.2 Effect of Probiotic Administration on Improving Emotional and Exploratory Behaviors Induced by Light–Dark Cycle Disruption

Figure 4B illustrates that rats in the control and L. Rh + LDA groups had significantly more time of mobility within the central area of the OF compared to the LDA group (*p* < 0.05). The results from the EPM test also show that the rats in the LDA group spent more freezing time in the CA than those in the control group, L. Rh + LDA, and L. Rh + L. De + LDA, and this difference between the LDA group and the other three mentioned groups was significant (*p* < 0.05) (Figure 5A).

In addition, the rats of the LDA group visited the CA more times than the other groups, with significant differences evident in Figure 5B.

It was also observed that the LDA group had lower mobile time in the OA compared to the groups that received probiotics, with the number of significant differences specified in Figure 5E.

As depicted in Figure 5C, no significant differences in distance traveled in the OA were observed between the groups receiving probiotics and the control group, suggesting that their behaviors were closely aligned.

In summary, these results indicate that the administration of *L. rhamnosus* and *L. delbrueckii* probiotics may contribute to reducing anxiety levels and enhancing exploratory behaviors in rats.

### 3.3 Comparison of the Effects of L. rhamnosus and L. delbrueckii on Emotional and Exploratory Behaviors

Analysis of the graphs obtained from the OF and EPM tests indicates that the administration of two probiotics, *L. rhamnosus* and *L. delbrueckii*, can partially ameliorate the effects of light–dark cycle disturbance on the anxiety and exploratory behaviors of rats. The contribution of *L. rhamnosus* appears to be more pronounced than that of *L. delbrueckii*. This assertion is supported by the data presented in Figure 5D, which shows a significant difference in average speed between the L. Rh + LDA and L. De + LDA groups (*p* < 0.05). This finding suggests that the administration of *L. rhamnosus* significantly enhances the rats' anxiety levels and exploratory behaviors compared to those receiving *L. delbrueckii*. Furthermore, Figure 4B from the OF test reveals a significant difference in the time spent in mobility within the center of the arena between the LDA and L. Rh + LDA groups (*p* < 0.05). In contrast, no significant difference is observed between the LDA and L. De + LDA groups regarding this parameter (table 5).

Also, in Figure 4B of the OF experiment (time mobile parameter), there is a significant difference between the rats of the LDA and L. Rh + LDA groups (*p* < 0.05), but this difference between the LDA and L. De + LDA groups was not significant.

### 3.4 Changing the Intestinal Microbiota by Administering Probiotics

In this study, a qualitative PCR molecular test was conducted to confirm alterations in the intestinal microbiota of rats. To perform this test, feces samples from rats were collected on the first day and again after thirty days. The results obtained from the PCR test of DNA extracted from rat feces are presented in Table 4.

**TABLE 4:** Results obtained from the qualitative PCR test.

Group	Test result	Number in Figure 6
L. Rh + LDA (before intervention)	−	1
L. De + LDA (before intervention)	−	2
L. Rh + LDA (after intervention)	+	3
L. De + LDA (after intervention)	+	4

*Note*: Positivity in this table refers to the presence of a band in the image, indicating the desired bacteria in the sample. In Figure 6, you can observe the presence of these bands.

## 4 Discussion

In a prior study, the effects of *Lactobacillus brevis* (ProGA28) on stress‐related changes were examined, demonstrating its potential to modulate the nervous system and affect motor responses in anxiety tests on Wistar rats (Lai et al. 2022). In our current study, we have explored the effects of two other *Lactobacillus* species, *L. rhamnosus* and *L. delbrueckii*, using behavioral and molecular assessments. Our findings suggest that these probiotics effectively improve disrupted emotional and exploratory behaviors following light–dark cycle alteration and alter the gut microbiota in rats. It is important to note that while universally established guidelines for light–dark cycle alterations in animal studies are lacking, the validity of using extended light cycles or reduced dark phases to investigate the effects of circadian misalignment is supported by previous research. For instance, studies have shown that disruptions in circadian rhythms caused by light–dark cycle changes can result in various health outcomes (Baron and Reid 2014). Moreover, exposure to light at night has been reported to disrupt circadian rhythms and affect physiological and behavioral processes (Meléndez‐Fernández et al. 2023). These findings form the basis for adopting a 20‐h light and 4‐h dark cycle in our study, allowing us to examine the effects of such disruptions on anxiety‐related behaviors.

Research by Schmidt et al. (2024) and Lin et al. (2022) examined the effects of *L. rhamnosus and L. delbrueckii, respectively*, in mice subjected to a high‐fat diet, with both studies demonstrating their anti‐anxiety effects. Additionally, a study from 2018 highlighted the impact of nighttime light exposure on laboratory animals (Emmer et al. 2018). Collectively, these studies affirm the significant influence of light on the physiology and behavior of laboratory animals, supporting the beneficial roles of *L. rhamnosus* and *L. delbrueckii* in alleviating anxiety behaviors (Olorocisimo et al. 2023).

In this study, we evaluated the effects of administering *L. rhamnosus* and *L. delbrueckii* on exploratory and anxiety behaviors in male Wistar rats subjected to a 30‐day light–dark cycle alteration. Our results indicate that such alterations can lead to anxiety and exploratory behavior disorders, while the administration of *lactobacilli*, particularly *L. rhamnosus*, significantly reduced anxiety and stress, enhancing exploratory activity. Behavioral tests, including the EPM and OF tests, were used to evaluate these behaviors. In the EPM test, rats spending more time in the OA exhibited lower stress levels. Conversely, in the OF test, rats spending more time in the center of the arena exhibited lower anxiety levels. Additionally, greater distances traveled indicated increased exploratory behavior. The OF test results revealed that rats exposed to 30 days of light–dark cycle disturbances experienced heightened stress levels. Specifically, the LDA group traveled shorter distances and spent less time in the center than the control group (Figure 4C,A). In the EPM test, rats in the LDA group exhibited reduced average speed, mean of visits, and time mobile in the OA compared to other groups, indicating decreased exploratory behavior and increased anxiety levels (Figure 5D–F). In contrast, the probiotics‐treated groups showed significant improvements in these behaviors. According to the OF test results, both the control group and the group administered *L. rhamnosus* spent significantly more time mobile in the center of the arena than the LDA group. In the EPM test, probiotic‐treated groups remained mobile for longer durations in the OA compared to the LDA group (Figure 5E). Overall, our findings suggest that both probiotics effectively enhanced exploratory behaviors and promoted relaxation in rats subjected to light–dark cycle alteration. However, as indicated in the results section and supported by statistical analyses, *L. rhamnosus* had more pronounced anti‐anxiety effects than *L. delbrueckii*; specifically, there were significant differences in average speed observed between the L. De + LDA and L. Rh + LDA groups (Figure 5D).

Some graphs showed, the group that received *L. rhamnosus* (L. Rh + LDA) exhibited lower levels of anxiety and enhanced exploratory behaviors compared to the group that received both probiotics (L. Rh + L. De + LDA). Conversely, the group receiving only *L. delbrueckii* (L. De + LDA) showed reduced stress compared to the combined group (L. Rh + L. De + LDA); however, these differences were not statistically significant (Table 5).

**TABLE 5:** In the control, LDA, PBS, L. Rh, L. De, and L. Rh + L. De groups, indicators were obtained in behavioral tests, OPT, and EPM.

Parameters / Groups		Control	LDA	PBS	L. Rh	L. De	L. Rh + L. De
**OF Test**	**Center: time (sec)**	9.79 ± 0.8	2.10 ± 0.8	3.61 ± 1.4	5.57 ± 2.1	4.24 ± 1.3	5.79±1.6
	**Center: distance (m)**	1.89 ± 0.2	0.35 ± 0.2	0.60 ± 0.3	1.36 ± 0.4	1.04 ± 0.4	0.75 ± 0.2
	**Center time mobile (sec)**	9.74 ± 0.8	2.10 ± 0.8	3.26 ± 1.4	9.97 ± 2.3	5.47 ± 2.2	5.24 ± 2.1
**EPM Test**	**Open Arms: av. Speed (m/s)**	0.054±0.004	0.025±0.001	0.033±0.004	0.080±0.029	0.038±0.003	0.025±0.004
	**Open Arms: distance (m)**	3.09 ± 0.3	1.39 ± 0.1	1.28 ± 0.3	2.23 ± 0.5	2.39 ± 0.4	2.68 ± 0.6
	**Open Arms: mean visit**	7 ± 0.7	4 ± 0.4	5 ± 0.9	8 ± 1.3	6 ± 1	5 ± 0.8
	**Open Arms: time mobile (sec)**	26.86 ± 2.2	10.51 ± 1	18.26 ± 2.4	29.23 ± 3.2	23.74 ± 3.4	27.39 ± 2.2
	**Closed arms: mean visit**	9 ± 0.7	23 ± 1.8	14 ± 1.5	11 ± 1.6	12 ± 1.3	14 ± 1.9
	**Closed arms: time freezing (sec)**	23.64 ± 3.7	50.57 ± 9.2	30.49 ± 4.5	24.89 ± 4.3	25.59 ± 4.1	27.66 ± 5.2

*Note*: Data are shown as mean ± SEM.

Another noteworthy finding is the impact of gavage on inducing anxiety behaviors in rats. This effect is particularly evident in the OF test, where time spent and distance traveled in the center of the arena were analyzed. The results indicate a significant difference between the PBS + LDA group and the control group (*p* < 0.05) (Figure 4A,C). In the EPM test, the distance traveled by the PBS + LDA group in the OA compared to the control group, as well as their mobility time in the OA compared to the L. Rh + LDA group, was significant (*p* < 0.05). These findings highlight meaningful differences in exploratory behavior. However, administration of probiotics, particularly *L. rhamnosus*, not only improved anxiety levels caused by light–dark cycle disruption but also largely mitigated gavage‐induced stress. For instance, in Figure 5E, the *L. rhamnosus* group spent significantly more mobility time in the OA compared to the LDA and PBS + LDA groups (*p* < 0.05). Given this observation, it is suggested that incorporating probiotics into the diet of rats might be more effective in alleviating these disorders, as it would eliminate the stress caused by gavage.

**FIGURE 6:**
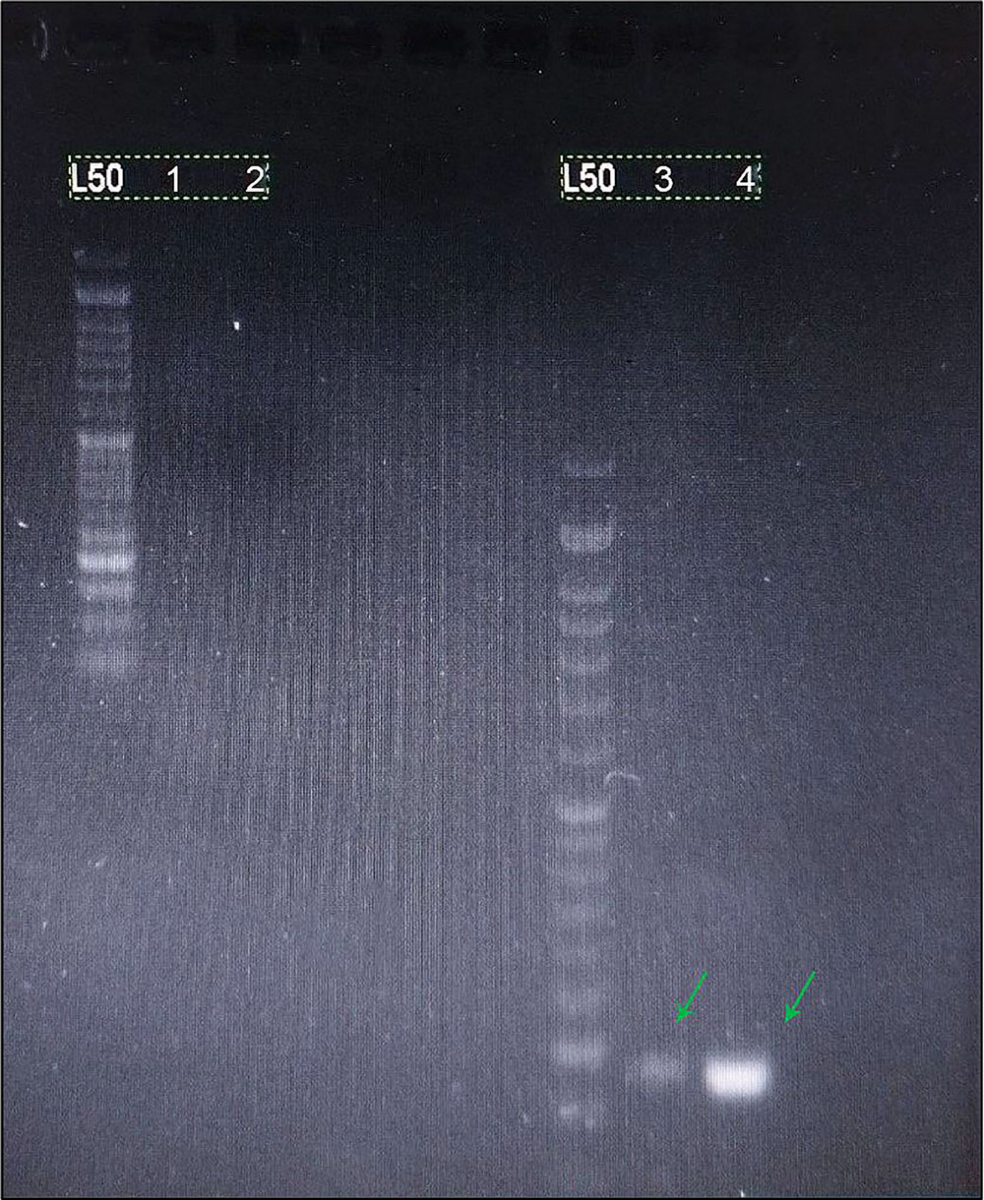
Appearance or absence of the band in different groups under the PCR test (According to the image, both primer sequences exhibit a 186 bp band due to their equal length [arrows]). L. Rh (*L. rhamnosus*). L. De (*L. delbrueckii*).

Showed minimal behavioral changes during the 30‐day period and tested negative for probiotics in the PCR test both at the start and end of the study. The molecular PCR test revealed that none of the target bacteria were naturally present in rat fecal samples at the beginning of the intervention. However, the presence of these bacteria in the fecal samples of the groups that received probiotics was confirmed, indicating the successful incorporation of these bacteria into the gut microbiota post‐intervention (Figure 6).

Although stress‐related hormones were not measured, previous studies suggest that increased light exposure during the night suppresses melatonin, potentially leading to sleep alterations and increased anxiety in rats (Nematullahi 2021; The National Institute for Occupational Safety and Health (NIOSH) 2023).

A growing body of evidence suggests the anxiolytic benefits of supplements made with other *Lactobacillus* species in rodents and humans (Yu et al. 2020). While anti‐anxiety effects are not limited to *L. rhamnosus*, studies have suggested similar beneficial effects for other *Lactobacillus* species, including *L. brevis* (Scholl et al. 2019), *L. helveticus* (Ait‐Belgnaoui et al. 2014), *L. reuteri* (Jang et al. 2019), and *L. plantarum* (Wu et al. 2021). It has also been said that consumption of *Lactobacillus* species regulates emotional behaviors and GABA receptor expression in rats through retrograde transport along the vagus nerve (Bravo et al. 2011).

The findings of this study have potential clinical significance. Modern lifestyles often involve disruptions to natural light–dark cycles due to shift work, jet lag, and prolonged exposure to artificial light. Such disruptions have been linked to increased incidence of anxiety, mood disorders, and other health complications (Walker et al. 2020).

Our study suggests that interventions using probiotics, particularly *L. rhamnosus* and *L. delbrueckii*, may alleviate these symptoms by modulating the gut–brain axis and reducing stress and anxiety. Importantly, probiotics offer a potentially safer and more natural adjuvant therapy for individuals suffering from circadian rhythm disorders, compared to pharmacological treatments that often come with side effects. While these findings are preliminary and require confirmation through human clinical trials, they provide a promising basis for the development of personalized probiotic interventions tailored to specific mental health conditions.

## 5 Conclusion

Our study demonstrated that rats exposed to a 30‐day disruption of the light–dark cycle exhibited increased anxiety and stress‐related behaviors.

This study highlights that the oral administration of *L. rhamnosus* and *L. delbrueckii* can improve emotional and exploratory behaviors under conditions caused by light–dark cycle disruption in male Wistar rats. It can be argued that these two *Lactobacillus* species modulate the intestinal microbiome and affect brain function through the gut–brain axis. Notably, the effect of *L. rhamnosus* in reducing anxiety behaviors and increasing exploratory activities was more pronounced than that of *L. delbrueckii*. Also, the growth and proliferation of these bacteria in the intestine were shown by molecular testing. Further studies focusing on sleep electrophysiological changes and endocrine responses are recommended.

## Author Contributions

**Farnaz Ghayour Babaei**: writing – original draft, methodology, investigation. **Ali Moghimi**: conceptualization, funding acquisition, writing – review and editing, project administration, supervision. **Ehsan Saburi**: conceptualization, writing – review and editing, methodology, supervision. **Ali Makhdoumi**: conceptualization, writing – review and editing, methodology. **Morteza Behnam Rasouli**: conceptualization, writing – review and editing, methodology.

## Ethics Statement

Animal care and all experimental procedures complied with the International and Iranian National Biomedical Ethics Committee (IR.UM.REC.1401.080).

## Consent

The authors have nothing to report.

## Conflicts of Interest

The authors declare no conflicts of interest.

## Peer Review

The peer review history for this article is available at https://publons.com/publon/10.1002/brb3.70814.

## Data Availability

The data that support the findings of this study are available from the corresponding author upon reasonable request.
